# Contributions to the Study of the Heart and Lungs

**Published:** 1888-03

**Authors:** 


**Contributions to the Study of the Heart and Lungs.** By JAMES R. LEAMING, M. D., Emeritus Professor of Diseases of the Chest and Physical Diagnosis, in the New York Polyclinic, etc. Pp. 300; 8vo. New York: E. B. Treat. 1887.

This work is composed of a number of monographs which the author has published at different times in medical journals and in medical society transactions. Those who are already familiar with Dr. Leaming's excellent methods of teaching, will highly value this collection of essays on his favorite subjects, while all physicians desiring to learn the peculiar views of the author will find no better method than by the perusal of this book. The publishers deserve special mention for the excellence of the typographical work.

